# Exercise-induced symptoms in young childhood cancer survivors

**DOI:** 10.1007/s11764-025-01877-3

**Published:** 2025-08-15

**Authors:** Maša Žarković, Carina Nigg, Christina Schindera, Myrofora Goutaki, Sonja Lüer, Marc Ansari, Claudia E. Kuehni

**Affiliations:** 1 Childhood Cancer Research Group, Institute of Social and Preventive Medicine, University of Bern, Mittelstrasse 43, 3012 Bern, Switzerland; 2 Graduate School for Health Sciences, University of Bern, Bern, Switzerland; 3 Division of Pediatric Oncology/Hematology, University Children’s Hospital Basel, Basel, Switzerland; 4 Division of Pediatric Respiratory Medicine and Allergology, Department of Pediatrics, Inselspital, Bern University Hospital, Bern, Switzerland; 5 Division of Pediatric Hematology and Oncology, Department of Pediatrics, Inselspital, Bern University Hospital, Bern, Switzerland; 6 Division of Pediatric Oncology and Hematology, Department of Women, Child and Adolescent, University Hospital of Geneva, Geneva, Switzerland; 7 CANSEARCH Research Platform for Pediatric Oncology and Hematology, Department of Pediatrics, Gynecology and Obstetrics, Faculty of Medicine, University of Geneva, Geneva, Switzerland

**Keywords:** Childhood cancer survivor, Respiratory health, Physical activity, Dyspnea

## Abstract

**Purpose:**

Exercise-induced symptoms (EIS) are common in children and understudied in childhood cancer survivors (CCS). We assessed the prevalence of EIS in CCS and identified associated risk factors.

**Methods:**

We included CCS aged 6–21 years who were diagnosed with cancer ≥ 1 year before study entry, were treated with systemic anticancer treatment, chest surgery, radiotherapy, or hematopoietic stem cell transplantation, completed cancer treatment, and received pediatric oncology follow-up care. Participants completed a questionnaire on respiratory symptoms and lifestyle. To explore risk factors of EIS, we used multivariable logistic regression and calculated population attributable fractions (PAFs).

**Results:**

Of 196 participants (median age 14 years [IQR 10–17]), 46 (24%) reported EIS, including dyspnea (14%), cough (12%), and wheeze (7%). EIS were more common among females (OR 2.5, 95% CI 1.1–5.9), older survivors (OR 1.2 per year, 95% CI 1.1–1.3,), and those with obesity (OR 4.7, 95% CI 1.1–19.6), asthma (OR 10.1, 95% CI 3.3–31.2), and physical inactivity (OR 2.9, 95% CI 1.3–6.6). Chest-directed radiotherapy tended also to increase the risk (OR 2.4, 95% CI 0.6–9.2). Twenty percent of EIS were attributable to asthma, 18% to physical inactivity, and 7% to obesity, with a combined PAF of 44%.

**Conclusions:**

EIS affect one in four CCS and are primarily associated with risk factors common in the general population rather than cancer treatments.

**Implications for Cancer Survivors:**

Clinical investigation and management of common causes of EIS, particularly asthma, physical inactivity, and obesity, could reduce symptom burden and support long-term health in CCS.

**Supplementary Information:**

The online version contains supplementary material available at https://doi.org/10.1007/s11764-025-01877-3.

## Introduction

Exercise-induced respiratory symptoms (EIS) are common in children and adolescents and most often present as dyspnea or shortness of breath, wheeze, and cough [1]. The most common causes of EIS are asthma leading to exercise-induced bronchoconstriction, insufficient fitness levels, and obesity [2, 3]. In other cases, EIS may be a manifestation of an underlying pulmonary pathology such as dysfunctional breathing disorders or restrictive lung disease [3].

Childhood cancer survivors (CCS) represent a particularly vulnerable group for long-term pulmonary complications due to possible late effects of oncologic treatments [4]. Chest-directed radiotherapy, thoracic surgery, pulmotoxic chemotherapeutic agents (e.g., bleomycin, busulfan), and hematopoietic stem cell transplantation (HSCT) can lead to structural and functional lung damage, including interstitial lung disease and reduced pulmonary capacity [4, 5]. While such impairments may remain asymptomatic at rest, they can become clinically evident during exercise, when respiratory demand increases [6].

Despite these known risks, EIS in CCS have been poorly characterized. Most studies reported on isolated symptoms, primarily wheeze [7] or dyspnea [8, 9], rather than evaluating a broader spectrum of EIS. One study found a continued increase in the cumulative incidence of exercise-induced dyspnea over time since completion of treatment [9], which suggests a growing and persistent burden. Yet the underlying causes of EIS in this population remain largely unexplored. Since EIS can contribute to avoiding physical activity [10, 11], characterizing these symptoms in CCS is essential given the role physical activity can play in mitigating treatment-related late effects, supporting psychosocial reintegration, and improving cardiopulmonary health [12, 13]. We therefore investigated the prevalence of EIS, identified factors associated with EIS, and quantified the contributions of these factors to EIS using population attributable fractions.

## Methods

### Study design and population

We used cross-sectional data from the Swiss Childhood Cancer Survivor Study (SCCSS) FollowUp–Pulmo, a multicenter study of CCS attending pediatric oncology follow-up care in Switzerland. Detailed study procedures have been published previously [14]. Briefly, SCCSS FollowUp–Pulmo investigates lung health in CCS aged 6 to 21 years who were diagnosed with cancer according to the International Classification of Childhood Cancer (ICCC-3) [15] at least one year before study entry. Eligible participants completed cancer treatment including systemic anticancer treatments (chemotherapy, immunotherapy, targeted agents), HSCT, thoracic surgery, or radiation to areas that may affect the lungs. We excluded CCS who were in a palliative or relapsed situation and those who were treated only with surgery or radiotherapy not affecting the chest area. The study collects clinical data from medical records and assesses symptoms and lifestyle via a questionnaire. For this analysis, we included participants enrolled between June 2022 and October 2024 at three pediatric oncology centers: Bern, Basel, and Geneva.

### Outcome: exercise-induced symptoms

We obtained data on EIS from a questionnaire that included a set of questions on respiratory problems during exertion. For survivors younger than 14 years, parents completed the questionnaire, while those aged 14 years or older filled it in themselves. If respondents answered “yes” to the question “Does your child/Do you sometimes have breathing difficulties during exertion,” they were asked to specify the symptoms including dyspnea, cough, or wheeze. We coded EIS as a binary variable (yes/no) if participants reported at least one symptom. We also asked about activities during which EIS occurred (running short 50–100 m or long > 500 m distances, cycling uphill, strenuous sports such as basketball, soccer, swimming, or other) to help differentiate potential underlying causes. These activities were selected based on their association with different mechanisms of EIS such as asthma, deconditioning, and obesity as previously described in the literature [16]. English translations of the original German/French questions are available in Supplementary Table S1.

### Explanatory variables

#### Sociodemographic and lifestyle characteristics

We retrieved information on participants’ age, height, and weight from medical records at the clinical visit corresponding to the date of questionnaire completion. Body mass index (BMI) was calculated and categorized according to World Health Organization (WHO) *z*-score standards: underweight (< − 2 SD), normal weight (≥ − 2 SD to ≤  + 1 SD), overweight (> + 1 SD), and obese (> + 2 SD) [17]. Due to the small number of underweight participants, we combined them with the normal-weight group for analysis. To describe the population’s sociodemographic and behavioral context, we assessed parental education, migration background, and smoking behavior (active and passive) via the questionnaire. Migration background was considered positive if both parents were born outside of Switzerland, and negative if the participant and at least one parent were born in Switzerland. Parental education level was classified into three categories: primary (compulsory schooling only, ≤ 9 years), secondary (vocational training or upper secondary education), or tertiary (university or technical college education). Active smoking was assessed in CCS older than 14 years and passive smoking exposure in all participants. If participants engaged in recreational sports, they were asked to specify the type of sport, the number of hours per week spent on it, and if they had a medical condition limiting their physical activity. For further analysis, we classified participants as physically inactive if they did not report engaging in any recreational sports, presuming their reduced physical fitness level.

#### Cancer- and treatment-related characteristics

We extracted the following cancer- and treatment-related information from medical records: age at diagnosis, cancer diagnosis, time since diagnosis, chemotherapeutic agents, immune- and targeted therapies, HSCT (yes/no), thoracic surgery (yes/no), pulmotoxic radiotherapy (yes/no), and relapse status (yes/no). We defined pulmotoxic chemotherapy as having received busulfan, bleomycin, or nitrosoureas (carmustine, lomustine) [18]. Thoracic surgery included any procedure involving the lungs or chest wall, excluding central line placement [18]. Pulmotoxic radiotherapy was defined as radiation to the chest (mantle, mediastinal, or whole lung fields), abdomen (whole or any upper field), or total body irradiation [5, 18]. We also extracted pulmonary diseases from medical records. Asthma was coded as “yes” if participants reported having asthma or using asthma medication in the questionnaire or had a current diagnosis of asthma in their medical records.

### Statistical analysis

We used mean and standard deviation (SD) or median and interquartile range (IQR) for continuous variables and frequencies and percentages for categorical variables to describe sociodemographic, lifestyle, and clinical characteristics. To identify factors associated with EIS, we performed a multivariable logistic regression. We selected a priori exposure variables based on clinical plausibility and existing literature [1, 5, 6], as conceptualized in Supplementary Fig. S1. These included age, sex, BMI categories, time since diagnosis, treatment exposures (pulmotoxic chemotherapy, thoracic surgery, HSCT, and pulmotoxic radiotherapy), asthma, and physical inactivity. To estimate the potential impact of these factors, we calculated population attributable fractions (PAFs), using the *punaf* command in Stata [19]. PAFs were derived from the fully adjusted logistic regression model and represent the proportion of EIS cases that might be prevented if these exposures were eliminated or effectively managed at the population level, assuming a causal relationship [20, 21]. We used Stata (Version 16.1, Stata Corporation, Austin, TX) for all analyses.

## Results

### Characteristics of the study population

Of 218 eligible survivors who attended pediatric oncology follow-up care in participating centers, 196 filled in the questionnaire (response rate 90%, Supplementary Fig. S2). More than half (59%) were male (Table 1). Median age at the time of the study was 14 years (IQR 10–17), median age at diagnosis was 5 years (IQR 3–9), and median time since diagnosis was 7 years (IQR 4–10). The most common diagnoses were leukemia (52%), lymphoma (10%), and neuroblastoma (9%). Nineteen participants (10%) had pulmotoxic chemotherapy, 25 (13%) pulmotoxic radiotherapy, 16 (8%) thoracic surgery, and 20 (10%) HSCT. Seventeen (8%) were obese. Twenty-two participants (11%) had asthma, and one was diagnosed with pulmonary graft-versus-host disease. CCS with EIS were older at study, older at diagnosis, engaged less in recreational sports, and more often had asthma compared to CCS without EIS.

**Table 1:** Characteristics of participating childhood cancer survivors with and without exercise-induced symptoms (*N* = 196)

	All CCS	CCS with EIS	CCS without EIS	*P*-value^a^
	*N* = 196	*N* = 46	*N* = 150	
Sociodemographic and lifestyle characteristics	*n *(%)^b^	*n* (%)^b^	*n* (%)^b^	
*Median age at study in years (IQR)*	14 (10–17)	15 (12–18)	13 (10–17)	0.035
*Age groups*				0.496
6–10	46 (24)	6 (13)	40 (26)	
11–14	71 (36)	16 (35)	55 (37)	
15–20	79 (40)	24 (52)	55 (37)	
*Sex (female)*	81 (41)	21 (46)	60 (40)	
*BMI categories*				0.212
Underweight	9 (5)	3 (7)	6 (4)	
Normal weight	121 (62)	23 (50)	98 (65)	
Overweight	49 (25)	14 (30)	35 (23)	
Obese	17 (8)	6 (13)	11 (7)	
*Migration background*^c^	59 (30)	10 (22)	49 (33)	0.158
*Parental education (highest level)*^d^				0.351
Primary or secondary education	82 (42)	22 (48)	60 (40)	
Tertiary education	102 (52)	21 (46)	81 (54)	
*Active smoking (n* = *92)*^e^	11 (12)	3 (11)	8 (12)	0.590
*Passive smoking*^f^	59 (30)	15 (33)	44 (29)	0.631
*Recreational sports*	145 (75)	25 (56)	120 (81)	0.001
Cancer- and treatment-related characteristics	*n *(%)^b^	*n* (%)^b^	*n* (%)^b^	
*Median age at diagnosis in years (IQR)*	5 (3–9)	7 (3–13)	4 (2–8)	0.002
*Median time since diagnosis in years (IQR)*	7 (4–10)	5 (4–9)	7 (5–10)	0.091
*Diagnosis (ICCC-3)*				0.384
Leukemia	102 (52)	23 (50)	79 (53)	
Lymphoma	20 (10)	6 (13)	14 (9)	
CNS tumor	11 (6)	2 (4)	9 (6)	
Neuroblastoma	18 (9)	3 (7)	15 (10)	
Renal tumor	15 (8)	3 (7)	12 (8)	
Soft tissue sarcoma	14 (7)	2 (4)	12 (8)	
Other tumor^g^	16 (8)	7 (15)	9 (6)	
*Treatment*				
Chemotherapy^h^	194 (99)	46 (100)	148 (99)	0.585
Pulmotoxic^i^	19 (10)	4 (9)	15 (10)	0.526
Pulmotoxic radiotherapy^j^	25 (13)	8 (18)	17 (11)	0.281
Thoracic surgery^k^	16 (8)	5 (11)	11 (7)	0.443
HSCT^l^	20 (10)	5 (11)	15 (10)	0.865
*Relapse*	15 (8)	3 (7)	12 (8)	0.515
Pulmonary disease	*n* (%)^b^	*n* (%)^b^	*n* (%)^b^	
Asthma	22 (11)	14 (30)	8 (5)	< 0.001
Pulmonary graft-versus-host disease	1 (0.5)	1 (2)	0 (0)	0.235

Abbreviations: *BMI* body mass index, *CCS* childhood cancer survivors, *CNS* central nervous system, *EIS* exercise-induced symptoms, *HSCT* hematopoietic stem cell transplantation, *ICCC-3* International Classification of Childhood Cancer - Third Edition, *IQR* interquartile range, *NA* not applicable

^a^*P*-value comparing childhood cancer survivors with and without exercise-induced symptoms derived from chi-squared or Fisher’s exact test for categorical and *t*-test for continuous variables

^b^Column percentages are given

^c^Migration background was considered positive if both parents were born outside of Switzerland and negative if the participant and at least one parent were born in Switzerland

^d^Data missing for 12 participants

^e^Question on active smoking was only asked for participants 14 years of age or older; data of five participants are missing

^f^Data missing for 11 participants

^g^Including retinoblastoma, hepatic, bone, germ cell tumors, other malignant epithelial neoplasms, malignant melanomas, other or unspecified malignant neoplasms, and Langerhans cell histiocytosis

^h^Any chemotherapy, targeted agents, or immunotherapy, alone or combined with other treatments

^i^Including busulfan, bleomycin, and nitrosoureas

^j^Radiotherapy involving chest, abdomen, spine, or total body irradiation alone or combined with other treatments

^k^Surgery involving thorax or lungs alone or combined with other treatments

^l^Any hematopoietic stem cell transplantation alone or combined with other treatments

While 51 CCS (26%, 95% confidence interval [CI] 20–33%) were physically inactive, most still engaged in recreational sports (74%, 95% CI 68–81%) (Supplementary Table S2). On average, participants spent 4.2 h per week on these activities. The types of sports that CCS engaged in are presented in Supplementary Fig. S3. Twenty-two participants (12%) reported having a medical condition that limited their physical activity, with musculoskeletal issues being the most common (Supplementary Table S2). Of those, 13 were physically inactive while nine still engaged in recreational sports.

### Prevalence of exercise-induced symptoms

One in four of 196 participants reported one or more EIS (24%, 95% CI 18–30%). Dyspnea was reported most commonly by 14% (95% CI 9–19%), followed by cough (12%, 95% CI 8–18%) and wheezing (7%, 95% CI 4–11%) (Table 2). Most participants reported symptoms when running long distances (> 500 m; 61%) or short distances (50–100 m; 52%).

**Table 2:** Exercise-induced symptoms in childhood cancer survivors

		*N* = 196		
		*n*	%	(95% CI)
Exercise-induced symptoms^a^		46	24	(18–30)
Dyspnea		27	14	(9–19)
Cough		24	12	(8–18)
Wheeze		13	7	(4–11)
*Activities during which symptoms occur *^b^		46		
Running				
Short distances (50–100 m)		24	52	(37–67)
Long distances (> 500 m)		28	61	(45–75)
Cycling uphill		18	39	(25–55)
Strenuous exercise (e.g., soccer or basketball)		21	46	(31–61)
Swimming		12	26	(14–41)
Other^c^		15	8	(4–12)

Abbreviation: *CI* confidence interval

^a^Defined as having reported at least one of the respiratory symptoms (dyspnea, cough, or wheeze). Participants may have reported more than one symptom

^b^Reported only by participants who had exercise-induced symptoms

^c^Self-reported situations such as climbing upstairs or hiking

### Factors associated with exercise-induced symptoms

**Fig. 1:**
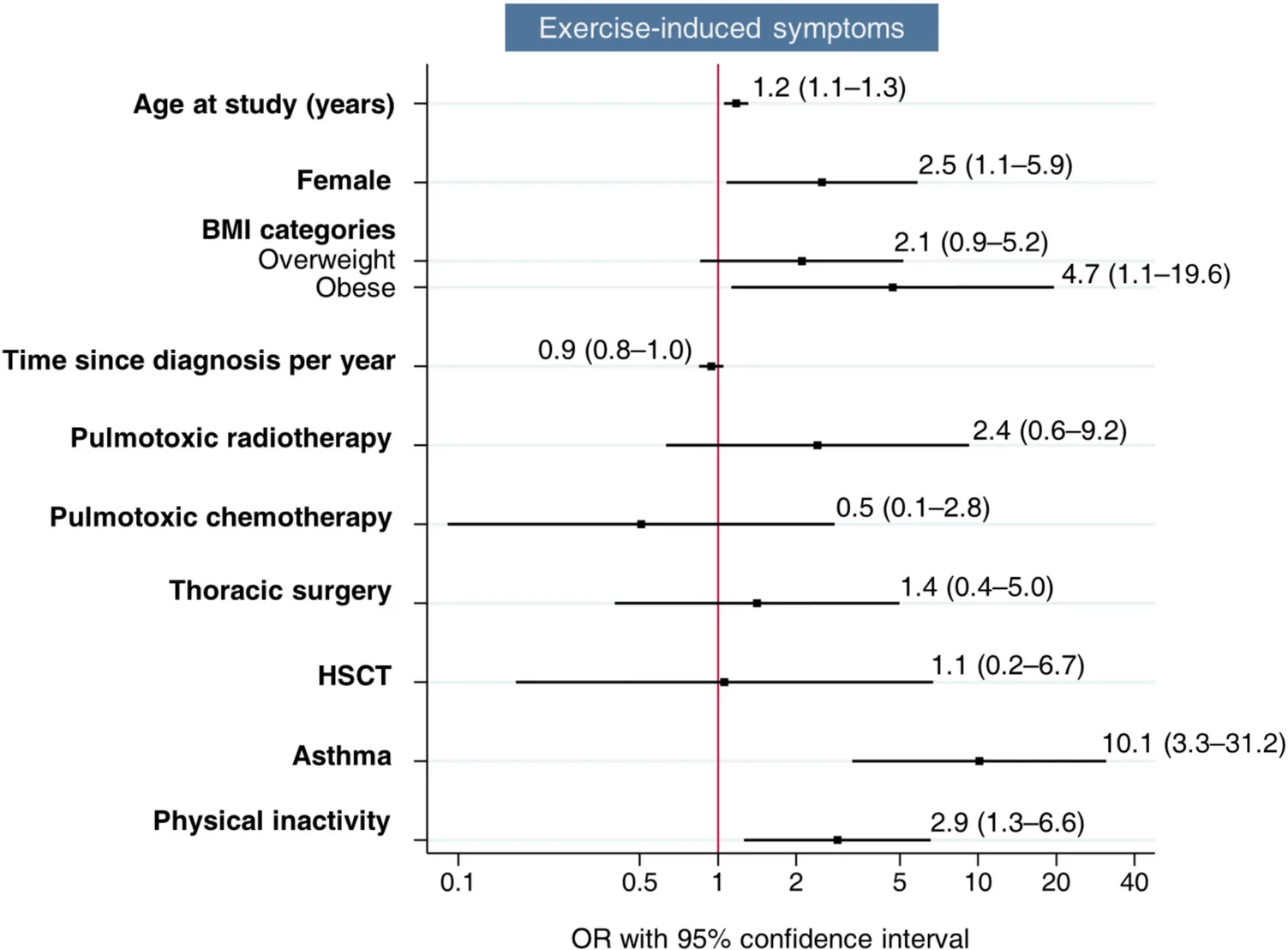
Factors associated with exercise-induced symptoms in childhood cancer survivors. Odds ratios from multivariable logistic regression adjusted for sociodemographic, lifestyle, and clinical characteristics. Abbreviations: HSCT, hematopoietic stem cell transplantation; OR, odds ratio

Females (OR 2.5, 95% CI 1.1–5.9), older survivors (odds ratio (OR) 1.2 per year of increase in age, 95% CI 1.1–1.3), obese survivors (OR 4.7, 95% CI 1.1–19.6, compared to normal weight), those with asthma (OR 10.1, 95% CI 3.3–31.2), and physically inactive survivors (OR 2.3, 95% CI 1.3–6.6) were more likely to experience EIS (Fig. 1, Supplementary Table S3). Survivors who received pulmotoxic radiotherapy (OR 2.4, 95% CI 0.6–9.2) showed a trend toward increased reporting of EIS. No associations were found with other treatment-related exposures. In adjusted analyses, an estimated 7% (95% CI − 0.5 to 15%) of EIS cases were attributable to obesity, 20% (95% CI 9–29%) to asthma, and 18% (95% CI 2–32%) to physical inactivity (Table 3). The joint PAF for obesity, asthma, and physical inactivity was 44% (95% CI 24–58%). PAFs for treatment-related exposures were small and had wide confidence intervals crossing zero.

**Table 3:** Estimated population attributable fractions and 95% confidence intervals for exposures associated with exercise-induced symptoms in childhood cancer survivors, based on adjusted logistic regression models

Exposure	PAF	95% CI
Overweight	11%	− 4–24%
Obesity	7%	− 0.5–15%
Pulmotoxic radiotherapy	7%	− 4–17%
Pulmotoxic chemotherapy	− 4%	− 16–6%
Thoracic surgery	2%	− 6–10%
HSCT	0.3%	− 12–11%
Asthma	20%	9–29%
Physical inactivity	18%	2–32%

PAFs were calculated from the adjusted logistic regression model applying the approach by Greenland and Drescher [20]

Abbreviations: *CI*, confidence interval, *HSCT*, hematopoietic stem cell transplantation, *PAF*, population attributable fraction

## Discussion

A substantial proportion of CCS—one in four—reported experiencing EIS. While survivors who received pulmotoxic radiotherapy showed a trend toward increased reporting of EIS, exposure to cancer treatment was not conclusively associated with them. However, a higher likelihood of EIS was independently associated with female sex, older age, obesity, asthma, and physical inactivity. Notably, 44% of these symptoms could be attributed to obesity, asthma, and physical inactivity—risk factors that are modifiable or treatable with targeted interventions similar to those available to the general population.

Few previous studies have examined EIS in childhood cancer survivors and mainly focused on single symptoms. For example, a USA-based questionnaire study of predominantly adult CCS reported a prevalence of 11% for exercise-induced dyspnea [9], while a study in pediatric acute lymphoblastic leukemia survivors found exertional dyspnea in 15% [22]. In a Swiss nationwide cohort of CCS aged 5–16 years, 6% reported exercise-induced wheeze [7]. Our findings of 14% reporting dyspnea and 7% reporting wheeze are generally consistent with these previous studies. Comparative data from the general population are limited, as most studies report EIS in the context of asthma or other chronic respiratory conditions. Nonetheless, cross-sectional surveys have found similar prevalence rates: 8–12% for exertional wheeze in Swiss schoolchildren aged 6–17 years [23], 14% for exercise-induced dyspnea among Swedish adolescents aged 12–13 years [24], and 11% for exercise-induced cough in French primary school children [25]. While our sample size precludes detailed age-stratified comparisons, the frequency of individual symptoms in CCS appears largely comparable to that in peers without a cancer history. However, the overall burden of EIS may be underestimated when only individual symptoms are assessed. In our study, one in four CCS (24%) reported at least one EIS, substantially higher than the prevalence of any single symptom alone. This highlights the need to ask about different symptoms during follow-up care since focus on individual symptoms may underestimate the overall burden. Such comprehensive screening during follow-up is also in line with current recommendations from the International Guideline Harmonization Group (IGHG), which emphasize the importance of remaining vigilant for symptom complaints to guide appropriate clinical evaluation [26].

Both asthma and obesity were significantly associated with EIS, reflecting patterns observed in the general pediatric population. Asthma is a well-established risk factor for EIS [6]. During physical exertion, increased ventilation cools and dries the airways, which can trigger bronchoconstriction and lead to symptoms such as wheezing, cough, and shortness of breath, particularly during prolonged aerobic activity [6, 16]. This aligns with our observation that the most commonly reported triggers were long- and short-distance running, activities known to provoke symptoms in individuals with poorly controlled asthma or low cardiorespiratory fitness [16]. Notably, effective asthma management can enable children to remain physically active and symptom-free [27]. Obesity was also an important risk factor and is known to impair respiratory mechanics through reductions in lung volumes, increased airway resistance, and greater work of breathing [28]. These physiological changes can contribute to the sensation of breathlessness during exercise, independent of underlying pulmonary disease [29]. In our population, an estimated 7% of EIS could be attributed to obesity and 20% to asthma. These findings emphasize the importance of recognizing and addressing these modifiable and treatable factors in both clinical assessments and survivorship care since targeted interventions could substantially reduce the symptom burden.

Physical inactivity was another major contributor to EIS . While 74% of CCS reported participation in recreational sports, this was lower than the 82–88% participation rate observed among Swiss schoolchildren in a national survey [30], suggesting that survivors may be at increased risk of inactivity. Several factors may explain this: parents may adopt protective attitudes that discourage sports participation, and children may underestimate their own capabilities or feel discouraged by early fatigue or physical limitations [31, 32]. Our findings nevertheless indicate that physical limitations are not always prohibitive; of the 12% of survivors who reported medical restrictions, 40% still engaged in sports. This suggests that with appropriate guidance and support, many CCS can remain active despite health challenges. Importantly, we estimated that 18% of EIS in our study could be attributed to physical inactivity, and the joint PAF for asthma, obesity, and inactivity was 44%. This means that nearly half of EIS cases could potentially be prevented or mitigated by addressing non-cancer-related factors.

The relationship between physical activity and EIS is likely bidirectional: symptoms may discourage exercise, while deconditioning from inactivity may worsen symptoms. However, evidence from the general population suggests that regular physical activity can reduce EIS, possibly by improving airway function and reducing inflammation [33]. Taken together, these results emphasize the potential for low-risk, modifiable interventions—including asthma management, weight control, and tailored exercise programs—to reduce EIS and improve overall health outcomes in CCS.

Sex and age, while not modifiable, may help identify CCS at higher risk for EIS. In our cohort, females were more likely than males to report EIS, which is consistent with findings from studies in healthy populations [34, 35]. This may reflect anatomical differences since females generally have smaller airways and lower lung volumes relative to body size, which increases the work of breathing and the likelihood of expiratory flow limitation during exertion [36]. We also found that older age was associated with higher EIS prevalence, which is in line with previous research suggesting that adolescents may engage in more strenuous physical activity and be more aware of their symptoms [37, 38]. These findings suggest that physiological and perceptual factors may contribute to symptom burden and should be considered in risk stratification and individualized follow-up care.

Regarding treatment-related factors, we did not find clear associations between pulmotoxic therapies and EIS. However, there was a tendency toward increased risk among CCS who received pulmotoxic radiotherapy. This aligns with findings from a study in adult CCS in which chest-directed radiotherapy was associated with higher rates of exercise-induced dyspnea, and the cumulative incidence continued to rise even decades after treatment [9]. Radiation-induced lung injury can lead to progressive parenchymal and interstitial changes, with studies suggesting these effects may worsen with age even if they remain clinically silent during childhood or adolescence [39, 40]. In our cohort, the median time since diagnosis was 7 years, which may be insufficient for these late effects to become fully apparent. Additionally, small numbers of survivors exposed to specific treatments may have limited our ability to detect statistically significant associations. Future research should include larger, longitudinal studies with long follow-up to better characterize the long-term effect of cancer therapies on EIS.

A key strength of our study is its high participation rate (90%), supporting the generalizability of our findings to CCS with similar cancer and treatment profiles. While treatment protocols and late effects are largely comparable across high-income countries, differences in population health behaviors, symptom perception, and background prevalence of conditions such as asthma or obesity may influence the prevalence or strength of associations in other settings [41, 42]. By assessing multiple EIS, we captured a more comprehensive picture of symptom prevalence than studies focusing on single symptoms. However, the cross-sectional design limits causal inference, and our findings should be interpreted as associative rather than causal. Reliance on self-reported data may introduce recall or reporting bias, leading to symptom misclassification due to differences in symptom perception, which can attenuate associations and reduce estimate precision. The relatively small number of EIS cases limited statistical power for subgroup analyses by treatment or diagnosis, which could be addressed in future studies by pooling data from larger national or international cohorts. Although we identified several modifiable contributors to EIS, nearly half of the symptom burden remained unexplained. Future research should explore additional factors such as undiagnosed cardiopulmonary disease, neuromuscular weakness, and psychological contributors like fatigue and anxiety, which may influence symptom perception. While self-reported symptoms offer important insight into survivors’ lived experiences, the lack of concurrent objective assessments, such as an exercise challenge test or echocardiographic data, limits our ability to directly attribute symptoms to underlying cardiopulmonary impairment. Future studies should ideally integrate objective measures to more comprehensively evaluate the mechanisms of exertional symptoms. These may include cardiopulmonary exercise testing, wearable accelerometers to quantify activity levels and intensities, and symptom diaries to capture the timing and context of symptoms, which could help clarify underlying mechanisms and guide targeted interventions [43, 44].

In summary, we found that a median of 7 years after cancer diagnosis EIS affects a substantial proportion of CCS, and in our cohort, nearly half of these symptoms could be attributed to modifiable or treatable risk factors, including asthma, obesity, and physical inactivity. Clinicians should be aware of these symptoms and first assess common, treatable causes before attributing them to late effects of cancer treatment. Early identification and targeted intervention for modifiable contributors, such as optimized asthma management, physical activity programs, and behavioral or nutritional counseling to promote healthy weight, could help reduce symptom burden, support physical activity, and improve long-term health outcomes in CCS [45, 46].

## Supplementary Information

Below is the link to the electronic supplementary material.

Supplementary file1 (DOCX 149 KB)

## Data Availability

No datasets were generated or analysed during the current study.
