# Supplementary material for: Exercise-induced symptoms in young childhood cancer survivors
Source: J Cancer Surviv. 2025 Aug 15;20(5):1894–902. doi: 10.1007/s11764-025-01877-3 (PMC13723604; doi:10.1007/s11764-025-01877-3)
Supplement: Supplementary file 1 [file 11764_2025_1877_MOESM1_ESM.docx]

# Supplementary Information

# Exercise-induced symptoms in young childhood cancer survivors

Maša Žarković^1,2^, Carina Nigg^1^, Christina Schindera^1,3^, Myrofora Goutaki^1,4^, Sonja Lüer^5^, Marc Ansari^6,7^, Claudia E Kuehni^1,5^

**Affiliations**

^1^ Institute of Social and Preventive Medicine, University of Bern, Switzerland

^2^ Graduate School for Health Sciences, University of Bern, Switzerland

^3^ Division of Paediatric Oncology/Haematology, University Children's Hospital Basel, University of Basel, Switzerland

^4^ Division of Paediatric Respiratory Medicine and Allergology, Department of Paediatrics, Inselspital, University Hospital, University of Bern, Switzerland.

^5^ Division of Pediatric Hematology and Oncology, Department of Pediatrics, Inselspital, Bern University Hospital, University of Bern, Switzerland

^6^ Division of Pediatric Oncology and Hematology, Department of Women, Child and Adolescent, University Hospital of Geneva, Switzerland

^7^ CANSEARCH Research Platform for Pediatric Oncology and Hematology, Department of Pediatrics, Gynecology and Obstetrics, Faculty of Medicine, University of Geneva, Switzerland

**Corresponding author**

Claudia Kuehni

Childhood Cancer Research Group, Institute of Social and Preventive Medicine, University of Bern

Mittelstrasse 43, 3012 Bern, Switzerland.

Email: [claudia.kuehni@unibe.ch](mailto:claudia.kuehni@unibe.ch)

Phone: +41 31 684 35 07

## Supplementary Figure S1. Conceptual framework showing possible predictors of exercise-induced symptoms and reduced physical activity as related to cancer treatments.

## Supplementary Table S1. English translations of original German/French questionnaire items on exercise-induced symptoms and recreational sports.

| **Variable** | | | **Question in questionnaire ^a^** | | | | |
| --- | --- | --- | --- | --- | --- | --- | --- |
| Exercise-induced symptoms (EIS) | | | Does your child / Do you sometimes have breathing difficulties on exertion? (yes/no) ^b^ | | | | |
|  | | |  | | | If yes, which?   - Dyspnea - Cough - Wheeze | |
| Activities triggering EIS | | | In which situation do the breathing difficulties occur? ^b^   - Running short 50-100m distances - Running long >500m distances - Cycling uphill - Strenuous sports (basketball, soccer, swimming) - Swimming - Other (free text) | | | | |
| Recreational sports | | | Does your child / Do you play sport regularly outside of school sports (e.g. soccer, dancing, gymnastics, judo, etc.)? (yes/no) | | | | |
|  |  | | | If yes, which and how many hours per week? (free text) | | |  |
| Medical condition limiting physical activity | | Does your child / Do you have a disability or illness that makes it difficult to play sport? (yes/no) | | | | |  |
|  | |  | | | If yes, which? (free text) | |  |

^a^ Questionnaires were filled in by parents of childhood cancer survivors if they were younger than 14 years, and by survivors themselves if they were older than 14 years.

^b^ Participants could select more than one answer.

Abbreviations: EIS, exercise-induced symptoms

## Supplementary Figure S2. Flow diagram of the study population.

Abbreviations: ICCC-3, International Classification of Childhood Cancer, third edition; HSCT, hematopoietic stem cell transplantation

## Supplementary Figure S3. Recreational sport types and frequencies among childhood cancer survivors stratified by sex. There can be multiple (1-3) recreational sport types per child.

Proportion of childhood cancer survivors (%)

|  | Females (n=81) | Males (n=115) |
| --- | --- | --- |
| Athletics | 0 | 1.73913 |
| Dancing | 19.7531 | 1.73913 |
| Horseback riding | 13.5802 | 0.869565 |
| Running | 0 | 2.6087 |
| Tennis | 1.23457 | 2.6087 |
| Skiing, snowboarding | 1.23457 | 3.47826 |
| Cycling | 3.7037 | 4.34783 |
| Gymnastics | 13.5802 | 6.08696 |
| Swimming | 11.1111 | 6.08696 |
| Hockey | 2.46914 | 7.82609 |
| Martial arts | 2.46914 | 8.69565 |
| Other ball games | 4.93827 | 13.0435 |
| Fitness | 7.40741 | 17.3913 |
| Soccer | 6.17284 | 27.8261 |

## Supplementary Table S2. Prevalence of recreational sport engagement and physical inactivity across age groups, and self-reported physical activity limitations.

|  |  | N=196 | | |
| --- | --- | --- | --- | --- |
|  |  | n | % | (95%CI) |
| **Recreational sports** | | 145 | 74 ^a^ | (68–81) |
| *Age categories, years* | |  |  |  |
|  | 6-10 | 35 | 76 ^b^ | (61–87) |
|  | 11-14 | 58 | 82 ^b^ | (71–90) |
|  | 15-20 | 52 | 66 ^b^ | (55–77) |
| *Average hours/week of recreational sports, mean, SD* | | 4.2 (2.9) | | |
| **Physical inactivity** | | 51 | 26 ^b^ | (20–33) |
| *Age categories, years* | |  |  |  |
|  | 6-10 | 11 | 24 ^b^ | (13–39) |
|  | 11-14 | 13 | 18 ^b^ | (10–29) |
|  | 15-20 | 27 | 34 ^b^ | (24–46) |
| **Physical activity limitations ^c,d^** | | 22 | 11 ^a^ | (7–17) |
|  | Musculoskeletal conditions ^e^ | 12 | 6 ^a^ | (3–10) |
|  | Cardiorespiratory conditions ^f^ | 3 | 2 ^a^ | (0–4) |
|  | Neurological conditions ^g^ | 3 | 2 ^a^ | (0–4) |
|  | Other ^h^ | 4 | 2 ^a^ | (0–5) |

^a^ Column percentages are presented.

^b^ Row percentages are presented.

^c^ Of 22 participants who reported physical activity limitations, 9 (41%) engaged in recreational sports.

^d^ Reporting a medical condition perceived as limiting physical activity.

^e^ Self-reported musculoskeletal conditions such as joint necrosis, shorter or missing leg, or using a wheelchair.

^f^ Self-reported cardiorespiratory conditions such as asthma or heart rhythm disorder.

^g^ Self-reported neurological conditions such as epilepsy.

^h^ Self-reported conditions such as unspecified problems after cancer disease.

## Supplementary Table S3. Association between exercise-induced symptoms and sociodemographic, lifestyle, and clinical characteristics in childhood cancer survivors using multivariable logistic regression.

|  |  | Exercise-induced symptoms | | |
| --- | --- | --- | --- | --- |
|  |  | OR | (95% CI) | *P-value* ^a^ |
| *Age at study (years)* | | 1.2 | (1.1-1.3) | 0.004 |
| *Sex* | |  |  | 0.033 |
|  | Male | Ref. |  |  |
|  | Female | 2.5 | (1.1-5.9) |  |
| *BMI categories* | |  |  | 0.062 |
|  | Normal weight ^b^ | Ref. |  |  |
|  | Overweight | 2.1 | (0.9-5.2) |  |
|  | Obese | 4.7 | (1.1-19.6) |  |
| *Time since diagnosis per year* | | 0.9 | (0.8-1.0) | 0.275 |
| *Pulmotoxic radiotherapy ^c^* | |  |  | 0.199 |
|  | No | Ref. |  |  |
|  | Yes | 2.4 | (0.6-9.2) |  |
| *Pulmotoxic chemotherapy ^d^* | |  |  | 0.437 |
|  | No | Ref. |  |  |
|  | Yes | 0.5 | (0.1-2.8) |  |
| *Thoracic surgery ^e^* | |  |  | 0.593 |
|  | No | Ref. |  |  |
|  | Yes | 1.4 | (0.4-5.0) |  |
| *HSCT ^f^* | |  |  | 0.954 |
|  | No | Ref. |  |  |
|  | Yes | 1.1 | (0.2-6.7) |  |
| *Asthma* | |  |  | <0.001 |
|  | No | Ref. |  |  |
|  | Yes | 10.1 | (3.3-31.2) |  |
| *Physical inactivity* | |  |  | 0.012 |
|  | No | Ref. |  |  |
|  | Yes | 2.9 | (1.3-6.6) |  |

^a^ P-value calculated from the Wald test.

^b^ Including underweight participants.

^c^ Radiotherapy involving chest, abdomen, spine, or total body irradiation.

^d^ Including busulfan, bleomycin, and nitrosoureas.

^e^ Surgery to the chest or lung (not including central line placement).

^f^ Including autologous and allogeneic hematopoietic stem cell transplantation.

Abbreviations: HSCT, hematopoietic stem cell transplantation; OR, odds ratio; CI, confidence interval.
